# Radio-Therapy

**Published:** 1905-05-27

**Authors:** 


### RADIO-THERAPY.

(*Continued from page* 139.)

Geo. Dock[16] classifies the reported cases of leukæmia treated with the rays as follows:—Two cases of acute lymphatic form died; two subacute cases died, though one was much improved. Of five chronic lymphatic forms one has died, the other four are greatly improved. Of 21 cases of mixed-celled, myelogenous, or spleno-medullary types, 10 were not detailed enough to be useful, and in the remaining 11 the leucocytes fell to near the normal.

Arthur Holding,[17] again, states that of 25 cases of spleno-medullary leukæmia reported treated by $x$-rays, eight were cured symptomatically, 15 were improved, and two were unimproved or dead. Of eight cases of lymphatic leukæmia reported as similarly treated, none were cured, three improved, and five unimproved or dead. Of 22 cases of pseudo-leukæmia, six were cured symptomatically, 13 improved, and three unimproved or dead.

Ledingham and McKerron,[18] in a very interesting and exhaustive analysis of the reported cases of the $x$-ray treatment of leukæmia, sum up their report with the remarks that all the patients suffering from myelogenous leukæmia have experienced under $x$-ray treatment remarkable improvement, both in the objective and subjective symptoms. They consider the question of the improvement being permanent cannot be entered into at present, owing to the tendency already noted in some cases towards relapse, and to the fact that many of the cases have been reported on the initial benefit being observed. There is abundant evidence that the blood change is profound; there are only a few cases recorded up to the present in which the leukæmia blood-picture has disappeared entirely. In the majority of cases the white cells have fallen to an approximately normal number, while the fall has been accompanied by a great diminution in the myelocyte percentage, and a corresponding rise in the polynuclear percentage. The red cells have almost invariably risen to a high level. In lymphatic leukæmia the treatment is only fairly satisfactory, however; and as an explanation for this they offer the suggestion made by Capps and Smith, who considered that while the chronic form of lymphatic leukæmia responds to the $x$-rays even more promptly than the spleno-myelogenous type, the acute and subacute varieties may not be so influenced. Further explanations may be attempted, but are only matters of conjecture. While some suggests an antiparasitic action on the part of the rays, Heinecke has found that their use on animals is attended by a disintegration of lymphocytes in the spleen and a destruction of the follicles of that organ and of the lymphoid structures and bone-marrow. The latter observer explains the lessening of the lymph glands and spleen in this way. Mosse and Milchner, in criticising Heinecke's statement as to the effect on the bone-marrow, state that the red cells, nucleated and non-nucleated, were unaffected. These observers accordingly grant the rays a place in leukæmia but not in pernicious anæmia. This the authors bear out in the observations they publish on a case of their own, but warn against attributing variations in the number of red cells entirely to the action of the rays; for in their case the red cells were found to vary considerably before $x$-ray treatment was commenced, a drop of 500,000 or even 1,000,000 in two days being even a marked feature without any therapeutic measures to account for it. No changes have been observed in normal blood corpuscles in salt solution under the $x$-rays (Joachim and Kurpjuweit, Mosse and Milchner), and it may be that all the bone-marrow of the body should be subjected to the influence of these rays. It may be that, as Lépine and Boulad have shown enzymes to be influenced by the rays, that Ehrlich's supposed chemiotactic enzyme in myelæmia may exist and be altered. Again, Schwarz has shown that radium has a destructive action upon the lecithin of an egg; and as the presence of lecithin bears so important relationship to the development of the cell (Hoppe-Seyler) its disintegration might prove fatal to further cell development.

For the treatment of lupus, Norman Walker[9] suggests using a plaster of oxide of uranium prepared from one of the readily obtainable salts (*e.g.* nitrate) incorporated with rosin or beeswax. It should be spread on leather and covered with waxed paper gummed at the edges. Such a plaster may be left on for 24 hours or more, and can be used without fear of too severe a reaction. Reaction has followed an application of over 24 hours on the face. His custom is to have it applied at nights only. It remains radio-active for months, and each plaster could be made for about a shilling. The success attending the use of this material he describes as very encouraging. Ledermann[20] follows Jesionek and Tapeiner in treating lupus with solutions of eosin. Applications were made several times a day, and it was shown that there was an elective action upon the lupus nodules. The effect is similar to pyrogallol, but is painless.

[16] Archiv Roent. Rays, March, 1905; Amer. Med., Dec. 24, 1904. [17] Med. Rec., Jan. 7, 1905. [18] Lancet, Jan. 14, 1905. [19] Scott. Med. and Surg. Jour., September, 1904. [20] Amer. Jour. Med. Sci., September, 1904.

